# The impact of cannabinoid receptor 1 absence on mouse liver mitochondria homeostasis: insight into mitochondrial unfolded protein response

**DOI:** 10.3389/fcell.2024.1464773

**Published:** 2024-10-24

**Authors:** Rosalba Senese, Giuseppe Petito, Elena Silvestri, Maria Ventriglia, Nicola Mosca, Nicoletta Potenza, Aniello Russo, Sara Falvo, Francesco Manfrevola, Gilda Cobellis, Teresa Chioccarelli, Veronica Porreca, Vincenza Grazia Mele, Rosanna Chianese, Pieter de Lange, Giulia Ricci, Federica Cioffi, Antonia Lanni

**Affiliations:** ^1^ Department of Environmental Biological and Pharmaceutical Sciences and Technologies, University of Campania “L. Vanvitelli”, Caserta, Italy; ^2^ Department of Sciences and Technologies, University of Sannio, Benevento, Italy; ^3^ Department of Experimental Medicine, University of Campania “Luigi Vanvitelli”, Naples, Italy

**Keywords:** cannabinoid receptor 1, mitochondrial quality control, homeostasis, mitochondrial unfolded protein response, respiratory chain supercomplexes, oxidative stress

## Abstract

**Introduction:**

The contribution of Cannabinoid type 1 receptor (CB1) in mitochondrial energy transduction mechanisms and mitochondrial activities awaits deeper investigations. Our study aims to assess the impact of CB1 absence on the mitochondrial compartment in the liver, focusing on both functional aspects and remodeling processes.

**Methods:**

We used CB1^−/−^ and CB1^+/+^ male mice. Cytochrome C Oxidase activity was determined polarographically. The expression and the activities of separated mitochondrial complexes and supercomplexes were performed by using Blue-Native Page, Western blotting and histochemical staining for in-gel activity. Key players of Mitochondrial Quality Control processes were measured using RT-qPCR and Western blotting. Liver fine sub-cellular ultrastructural features were analyzed by TEM analysis.

**Results and discussion:**

In the absence of CB1, several changes in the liver occur, including increased oxidative capacity, reduced complex I activity, enhanced complex IV activity, general upregulation of respiratory supercomplexes, as well as higher levels of oxidative stress. The mitochondria and cellular metabolism may be affected by these changes, increasing the risk of ROS-related damage. CB1^−/−^ mice show upregulation of mitochondrial fusion, fission and biogenesis processes which suggests a dynamic response to the absence of CB1. Furthermore, oxidative stress disturbs mitochondrial proteostasis, initiating the mitochondrial unfolded protein response (UPR^mt^). We noted heightened levels of pivotal enzymes responsible for maintaining mitochondrial integrity, along with heightened expression of molecular chaperones and transcription factors associated with cellular stress reactions. Additionally, our discoveries demonstrate a synchronized reaction to cellular stress, involving both UPR^mt^ and UPR^ER^ pathways.

## Graphical Abstract

**Figure:**
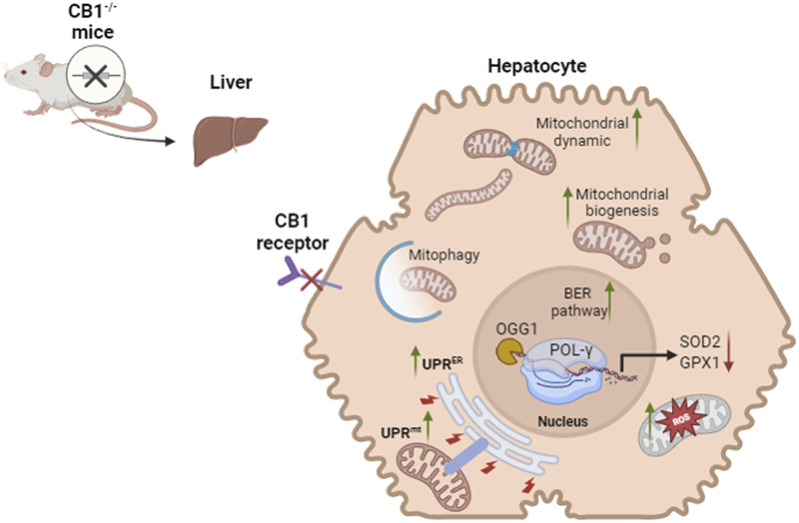

## 1 Introduction

The endocannabinoid system (ECS), comprising cannabinoid type 1 receptors (CB1) and type 2 receptors (CB2), their natural ligands termed endocannabinoids, and the enzymes responsible for their synthesis and degradation, is widely distributed throughout central and peripheral tissues (Pertwee, 2015). This system plays a pivotal role in regulating various physiological processes such as energy homeostasis, synaptic plasticity, and feeding behaviors (Lu and Mackie, 2016). CB1, in particular, has been extensively studied for its involvement in modulating central and peripheral processes to regulate energy metabolism. It seems very important, especially in contemporary society where obesity and metabolic syndrome have reached epidemic proportions due to an increasingly sedentary lifestyle and a diet high in processed food. Notably, hyperactivation of CB1 has been implicated in promoting metabolic processes leading to weight gain, lipogenesis, insulin resistance, and dyslipidemia (Cota et al., 2003; Di Marzo et al., 2001; Liu et al., 2012). Pharmacological inhibition or genetic deletion of CB1 has shown promise in ameliorating metabolic abnormalities in obese animals, leading to weight loss and improving insulin sensitivity (Jourdan et al., 2010). The benefits of ECS activity are greater when food is scarce. In contrast, ECS activity promotes obesity and metabolic syndrome when food is plentiful, suggesting a reactive homeostatic mechanism to optimize survival (Mazier et al., 2015; Nunn et al., 2010). It has been proposed that the increased ECS tone during metabolic syndrome may be considered a homeostatic protective mechanism induced by oxidative stress. Thus, the increased ECS activity might start as an attempt to compensate for metabolic dysfunctions, in the long term, it may exacerbate the metabolic syndrome (Nunn et al., 2010).

In addition to their central distribution, CB1 receptors are also widely expressed in peripheral tissues, including the liver (Mackie, 2005; Tam et al., 2011). Under normal physiological conditions, CB1 is not highly expressed in the liver. However, in pathological conditions such as obesity or hepatic steatosis induced by alcohol or nonalcoholic factors, CB1 expression is significantly upregulated, playing a pivotal role in hepatic insulin resistance, fibrosis, and lipogenesis (Busquets-García et al., 2022; Liu et al., 2012; Osei-Hyiaman et al., 2008). Remarkably, animals lacking hepatic CB1 do not develop dyslipidemia, insulin/leptin resistance, or steatosis when exposed to a high-fat diet (HFD) (Wang et al., 2021).

The liver, being a principal organ responsible for regulating metabolic homeostasis, relies heavily on the quality and quantity of hepatic mitochondria to perform metabolic activities efficiently. Hepatocytes harbor a large number of mitochondria to carry out various metabolic functions, primarily dependent on the integrity of Electron Transport Chain (ETC.) complexes and their organization into supercomplexes (Avram et al., 2022; Giacomello et al., 2020; Letts and Sazanov, 2017). These supramolecular arrangements play a crucial role in metabolic adaptation, responding to changes in the metabolic source of electrons and enhancing the efficiency of respiratory chain reactions (Kobayashi et al., 2023). In addition to energy production, mitochondria are also a significant source of Reactive Oxygen Species (ROS). Mitochondrial ROS, generated by the respiratory chain, play a central role in numerous cellular signaling pathways both within and outside the mitochondria (Finkel, 2012). However, excessive ROS production can lead to oxidative stress, characterized by an imbalance between mitochondrial ROS production and removal, ultimately affecting various cellular components, including mitochondrial DNA (mtDNA) (Bolisetty and Jaimes, 2013; Valko et al., 2007).

The CB1 plays an important role in mediating the biological effects of cannabinoids, whether they are derived from plants (phytocannabinoids), synthetically produced (synthetic cannabinoids), or naturally occurring in the body (endocannabinoids) (Blebea et al., 2024; Di Marzo and Piscitelli, 2015). Phytocannabinoids are phenolic compounds and can induce metabolic effects via CB receptors, but a lot of their actions appear to be independent of CB receptors, including direct effects on mitochondria (Nunn et al., 2010). Phytocannabinoids have been shown to affect mitochondrial function in various ways. The mitochondria, being central to cellular energy production, apoptosis regulation, and ROS balance, are directly influenced by phytocannabinoids like THC (Δ9-tetrahydrocannabinol) and CBD (cannabidiol) (Pagano et al., 2023; Podinic et al., 2024; Podinić et al., 2023; Rupprecht et al., 2022). The scientific literature offers many reviews and original research discussing the relationship between phytocannabinoids and mitochondria (Athanasiou et al., 2007; Bartova and Birmingham, 1976; Chan and Duncan, 2021; Fišar et al., 2014; Mato et al., 2010; Mould et al., 2021; 2021; Nunn et al., 2023; Olivas-Aguirre et al., 2019; Ryan et al., 2009). Since 1970, several studies have analyzed the effects of phytocannabinoids on mitochondrial function. The first studies were performed in the years ‘71 and ’72. These studies have been demostrated that THC could inhibit the ATPase activity of rat liver mitochondria and strongly affected rat liver mitochondria *in vitro*. At concentrations of 15–60 nmoles/mg of mitochondrial protein, THC uncoupled state IV respiration. It has been also demonstrated that THC could inhibit complex I and III of the ETC (Bartova and Birmingham, 1976). Bino and colleagues demonstrated that THC could alter the mitochondrial shape in response to the dose, which correlated with their respiratory rate (Bino et al., 1972). Research performed by another group confirmed that THC could induce mitochondrial swelling in several different tissues in rats (Banerji et al., 1985). According to Devane and colleagues, the brain contains cannabinoid receptors. Consequently, the endocannabinoid system was discovered, and phytocannabinoids and their research have increased (Devane et al., 1988).

In the 2000s, interest in the study between mitochondria and phytocannabinoids increased exponentially, suggesting that both THC and CBD can modulate mitochondrial function (Athanasiou et al., 2007; Sarafian et al., 2003; Whyte et al., 2010). THC is also known to disrupt neuronal mitochondrial function, affecting complexes I, II, and III, decreasing coupling, and enhancing ROS production (Wolff et al., 2015). Later Rimmerman and colleagues studied the effects of CBD on various mitochondrial functions in BV-2 microglial cells. They showed that CBD treatment led to a biphasic increase in intracellular calcium levels and changes in mitochondrial function and morphology leading to cell death. They proposed that CBD treatment induced mitochondrial swelling in a process involving VDAC1 and mitochondrial permeability transition pore (MPTP). These effects appeared to result from direct CBD-induced inhibition of the VDAC1 channel conductance (Rimmerman et al., 2013). Olivas-Aguirre and colleagues have demonstrated that CBD directly modulates mitochondrial calcium in lymphoblastic leukemic cells (Olivas-Aguirre et al., 2019). In addition, many compounds in plants can affect mitochondrial dynamics as well as mitochondrial function. Alistar V. W. Nunn and colleagues found that phytocannabinoids can affect mitochondrial dynamics (Nunn et al., 2020). Using MCF7 cancer cells, they showed that CBD has a dose-dependent impact on mitochondrial metabolism and morphology, and there is evidence that it induces oxidative stress at higher concentrations (Mould et al., 2021). Moreover, phytocannabinoids can influence mitochondrial dynamics, which involves the processes of fission and fusion that maintain mitochondrial health and adaptability (Malheiro et al., 2023; Walker et al., 2021).

In addition to the relationship between phytocannabinoids and mitochondria, several studies in the literature have also focused their attention on the involvement of the ECS in mitochondrial function. The ECS has evolved in a living organism to ensure the survival of the animal, and because of this, it interacts and modulates other systems, such as the mitochondria (Gomez et al., 2011; Howlett et al., 2010; Moreira et al., 2012; Nunn et al., 2012; Puighermanal et al., 2009; Velasco et al., 2005). Several studies suggested that THC, AEA, and HU210 could inhibit mitochondrial function. Catanzaro et al. have also shown that AEA increased mitochondrial swelling and reduced cytochrome C release associated with reduced membrane potential and increased membrane fluidity (Athanasiou et al., 2007; Catanzaro et al., 2009). As suggested by Nunn and colleagues, all the studies reported above support the idea that ECS modulates pathways that are known to affect mitochondrial activity, reinforcing the idea that all systems are interconnected to adapt to a variable environment. The fact that the ECS regulates several pathways that act on mitochondrial activity cannot be a coincidence. The results shown above, in particular, shed light on a multiphasic stimulus-adaptation response (Nunn et al., 2012).

To maintain proper mitochondrial and cellular function, cells employ various surveillance measures, including quality control mechanisms, to counteract dysregulated mitochondrial activity (Ni et al., 2015). Mechanisms for maintaining mitochondrial quality include mitochondrial biogenesis, fusion and fission, mitophagy, mtDNA repair, and the mitochondrial Unfolded Protein Response (UPR^mt^), all of which require accurate communication and coordination between the nuclear and mitochondrial genomes. Mitochondrial fusion and fission are crucial processes governing the morphology and function of the mitochondrial network. A mitochondrial fusion response may be seen as a response to the increased oxidative stress and serves several purposes (Adebayo et al., 2021; Al Ojaimi et al., 2022). While fusion promotes mitochondrial network integrity and functionality, it also creates a platform for efficient mitophagy (Shirihai et al., 2015; Twig et al., 2008). The UPR^mt^ functions as a signaling pathway, contributing to maintaining mitochondrial quality and ensuring the integrity of the mitochondrial proteome (Haynes and Ron, 2010). When misfolded proteins or incomplete complexes accumulate beyond the folding capacity, it disrupts proteostasis, causing damage and dysfunction within the organelle and the cell. While extensively studied in the Endoplasmic Reticulum (ER), recent findings suggest that similar signaling mechanisms operate in mitochondria, facilitating communication with the nucleus in response to proteostasis impairment (Cagin and Enriquez, 2015).

The UPR^mt^ is triggered by various types and levels of stress, particularly in conditions where unfolded or misfolded mitochondrial proteins accumulate and form aggregates (Haynes and Ron, 2010). It restores mitochondrial protein homeostasis and normalizes organelle function by decreasing protein levels, facilitating protein folding, or enhancing protein degradation (Fiorese and Haynes, 2017).

In mammals, the canonical UPR^mt^ signaling cascade comprises transcription factors such as C/EBP Homologous Protein (CHOP), Activating Transcription Factor 4 (ATF4), and Activating Transcription Factor 5 (ATF5), with ATF5 playing the central role as the main regulator (Fiorese et al., 2016; Zhao et al., 2002). Although ATF4 and CHOP have been reported to play a crucial role in UPR^mt^ regulation by promoting ATF5 transcription, the exact communication mechanism is not fully understood. UPR^mt^ activation begins with the translocation of these transcription factors into the nucleus, where they stimulate the transcription of proteases such as Caseinolytic mitochondrial matrix Peptidase Proteolytic subunit (CLPP) and Lon Peptidase 1 (LONP1), as well as Chaperones like Heat Shock Protein 60 (HSP60) and TNF Receptor-Associated Protein 1 (TRAP1), which constitute the core of restoring protein balance. They localize and operate within mitochondria in response to the accumulation of misfolded proteins or an imbalance between mitochondrial and nuclear encoded proteins within the organelle (Rd et al., 1996; Zhao et al., 2002). These components play a critical role in promoting protein folding and preventing protein aggregation across various cell types (Bukau et al., 2006).

Recent research indicates that UPR^mt^ can be activated under various conditions, including damage to, ETC., alterations in mitochondrial dynamics, deletion of mtDNA, and elevated levels of ROS (Muñoz-Carvajal and Sanhueza, 2020).

Similarly, to the UPR^mt^, the Unfolded Protein Response of the endoplasmic reticulum (UPR^ER^) maintains proteomic stability within the ER (Wang and Kaufman, 2012). Since mitochondria and the ER serve as primary centers for energy metabolism and protein synthesis in cells, it is likely that a synergistic mechanism exists between UPR^mt^ and UPR^ER^ to collectively combat stressors such as increased ROS levels. Mounting evidence suggests that the protein kinase RNA (PKR)-like Endoplasmic Reticulum Kinase (PERK) signaling pathway serves as a crucial node for coordinating UPR^mt^ and UPR^ER^ (Liu et al., 2014). The PERK pathway becomes activate in both UPR^mt^ and UPR^ER^, with its downstream molecules, including ATF4, CHOP, and ATF5, playing crucial functions.

As a result of metabolic stimuli and other changes within mitochondria, the activation of retrograde mitochondrial to nuclear signaling can lead to changes in nuclear gene expression. The activation of this signaling is essential to facilitate the reestablishment of homeostasis by refolding or removing unfolded or misfolded proteins (Arnould et al., 2015). Furthermore, it has been suggested that mitochondria-nucleus communication plays a crucial role in mitochondrial stress responses (Haynes et al., 2007; Haynes and Ron, 2010; Yoneda et al., 2004).

In recent years, accumulating evidence has indicated that the endocannabinoid system has the capacity to regulate both the integrity and functionality of mitochondria, thereby contributing to the maintenance of cellular energy homeostasis (Lipina et al., 2014). Recent research has elucidated the involvement of CB1 and its signaling pathways in various mitochondrial activities critical for regulating energy metabolism in both the brain and peripheral organs (Busquets-García et al., 2022; Pagano Zottola et al., 2022; Paszkiewicz et al., 2020; Senese et al., 2024). Given that CB1 mRNA levels in the liver are low under normal conditions, but increase in specific liver dysfunctions such as steatosis, studies have predominantly focused on obesogenic conditions such as HFD (Liu et al., 2012, p. 1; Wang et al., 2021). Moreover, although studies on CB1-deficient mice have enhanced our comprehension of CB1 signaling’ role in regulating liver metabolic functions, there remains a gap in our knowledge regarding the modulation of mitochondria, a dynamic cell compartment. It has also been shown cannabinoids disturb calcium homeostasis via multiple mechanisms, including stimulation of CB1 (Lauckner et al., 2005). In addition, while some studies have suggested that cannabinoid-induced increases in cytosolic calcium levels are a result of extracellular Ca2+ influx, others suggest that intracellular stores, such as the endoplasmic reticulum and mitochondria, may be responsible (Drysdale et al., 2006; Mato et al., 2010; Rossi et al., 2019; Ryan et al., 2009). The mechanism underlying the regulation of mitochondrial calcium by cannabinoids does not appear to depend on plasma membrane receptors but rather occurs through the direct targeting of mitochondria (Catanzaro et al., 2009; Ma et al., 2015; Olivas-Aguirre et al., 2019). Therefore, further investigations into the role of CB1 in regulating mitochondrial processes are essential for a deeper understanding of how this regulation maintains mitochondrial homeostasis by facilitating constant interaction among various response mechanisms.

In this context, our study aims to assess the impact of CB1 absence on liver mitochondria, focusing on both functional aspects and remodeling processes, utilizing a CB1^−/−^ male mouse model. Specifically, we investigate changes in respiratory capacity, respiratory chain complexes and supercomplexes, mitochondrial ROS production and antioxidant defenses. In addition, we evaluate MQC mechanisms, including biogenesis, dynamics, encompassing fusion and fission processes, mitophagy, and mtDNA repair. Furthermore, given the paucity of data on the relationship between CB1 activation/blockade and UPR^mt^, we explore the involvement of UPR^mt^ pathways in maintaining liver mitochondrial proteostasis and homeostasis in CB1^−/−^ mice.

## 2 Materials and methods

### 2.1 Animals and animal care

Mice (*Mus musculus*) genetically deleted for CB1 were provided by Prof. Ledent (Ledent et al., 1999). Male and female CB1 heterozygous (CB1^+/−^) mice have been maintained on a CD1 background (Charles River Laboratories, Lecco, Italy) to expand the colony, then used to generate adult CB1^+/+^ and CB1^−/−^ male mice. The animals were maintained in a temperature-controlled room at 22°C ± 2°C, under a 12 h dark/light cycle with a standard pellet diet and free access to water. Adult males (three to five months) were sacrificed and subjected to tissue collection. In detail, the animals were placed in a plexiglass chamber with 4% isoflurane (IsoVet, Piramal Healthcare, United Kingdom Limited) for 5 min. After that, when fully sedated as measured by a lack of heartbeat and active paw reflex, the animals were sacrificed by cervical dislocation. Liver tissue was rapidly removed, weighed and frozen in liquid nitrogen for molecular investigations, or fixed for morphological analyses. All animals received human care according to the criteria outlined in the Guide for the Care and Use of Laboratory Animals prepared by the National Academy of Sciences and published by the National Institutes of Health. Every effort was made to minimize animal pain and suffering. The minimum sample size (n = 5) was calculated using a G* Power Test, developed by the University of Dusseldorf (http://www.gpower.hhu.de/), required to get permission for *in vivo* experiments in Italy, suggested by the Legal Entity giving the permission. Experiments involving animals were approved by the Italian Ministry of Education and the Italian Ministry of Health, with authorization n°941/2016-PR issued on 10.10.2016.

### 2.2 Transmission Electron Microscopy (TEM) analysis

To evaluate liver fine sub-cellular ultrastructural features, CB1^+/+^ and CB1^−/−^ livers were minced in small pieces and fixed overnight in glutaraldehyde 2.5% (Electron Microscopy Science, Hatfield, PA, United States). After fixation with cacodylate buffer (pH 7.4) for at least 1 h, samples were post-fixed with 1% osmium tetroxide (OsO4) in 0.1 M cacodylate buffer, dehydrated in ethanol (Sigma-Aldrich, Milano, Italy, EU), and embedded in epoxy resin. Ultrathin sections (60 nm) obtained using an UC6 ultramicrotome (Leica, Wetzlar, Germany, EU) equipped with diamond knife (DiATOME US, Hatfield, PA, United States), were placed on copper grids (Electron microscopy science, Hat-field, PA, United States). Ultrathin sections were then treated with Uranyl acetate replacement stain (UAR - Electron Microscopy Science, Hatfield, PA, United States) and contrasted with lead hydroxide. Samples were studied using a 100 kV transmission electron micro-scope EM208S PHILIPS (FEI-Thermo Fisher, Waltham, MA, United States) equipped with the acquisition system Megaview III SIS camera (Olympus/EMSIS) and iTEM3/Radius software, version 2.1.

### 2.3 Measurement of H_2_O_2_ and 4-Hydroxynonenal (4-HNE) in liver samples

H_2_O_2_ was measured in liver samples using the hydrogen peroxide Assay Kit (Colorimetric/Fluorimetric) (Cat. no. 102500 Abcam). Liver tissues (40 mg) were homogenized rapidly in buffer, followed by centrifugation at 10,000xg at 4°C for 2–5 min, then the supernatant was collected for the deproteinization protocol. After deproteinization, we centrifuged the sample at 13,000xg for 15 min at 4°C and collected supernatant. For the deproteinization protocol, we used perchloric acid (PA) 4M, and ice cold potassium hydroxide (KOH) 2M. Subsequently, we prepared a master mix of the reaction mix (Assay Buffer, OxiRed Probe, Developer Solution V/HRP) and added 50 μL of the Reaction Mix and 50 μL of the sample. After incubation for 10 min at room temperature, fluorescence was measured at a microplate reader BioTek Sinergy H1 (Ex/Em = 535/587 nm) (Agilent, United States). The levels of 4-HNE in liver samples were measured using an ELISA kit (Cat. no. 287803 Abcam). Liver tissues (500 mg) were homogenized rapidly in buffer, followed by centrifugation at 5,000 × g at 4°C for 5 min, then the supernatant was collected. After loading the samples and standards into the plate, biotin-labeled antibodies and SABC were added. The absorbance is read at 450 nm with a microplate reader BioTek Sinergy H1 (Agilent, United States). The concentration of 4-HNE in the samples was determined by comparing the optical density to the standard curve.

### 2.4 Mitochondria isolation

Differential centrifugation was used to isolate mitochondria from the liver. An isolation medium consisting of 220 mM mannitol, 70 mM sucrose, 20 mM TRIS-HCl, 1 mM EDTA, and 5 mM EGTA (pH 7.4) supplemented with 0.1% BSA was used to gently homogenize tissue fragments using a Potter-Elvehjem homogenizer (Heidolph Instruments, Germany). The homogenate was centrifuged at 800 *g* for 10 min at 4°C. Subsequently, the supernatant was separated from the pellet and centrifuged at 3,000×g for 30 min at 4°C. For later use, mitochondrial pellets were washed twice, resuspended in an isolation medium, and kept on ice or at −80°C for later processing.

### 2.5 Determination of cytochrome oxidase activity (COX) in liver mitochondria

COX activity measurement was performed as described by (Petito et al., 2023). Briefly, aliquots of mitochondria were incubated for 30 min at 0°C after the addition of 1.0 mg/mL lubrol. COX activity was determined polarographically at 37°C by using the Oroboros 2k- Oxygraph system instrument (O2k, OROBOROS INSTRUMENTS, Inns-bruck, Austria). Mitochondrial homogenates were incubated with 30 μM Cytochrome C, 4 μM Rotenone, 0.5 mM Dinitrophenol, 10 mM Na-malonate, and 75 mM HEPES at pH 7.4 in 2 mL of the reaction medium. A substrate of 4 mM Na ascorbate with 0.3 mM of N,N,N′, N′-tetramethyl-p-phenylenediamine was added after 10 min to determine oxygen consumption. The auto-oxidation of the substrate was evaluated in parallel measurements in the absence of mitochondrial homogenate. Sample protein content was determined using Bio Rad’s DC method (Bio-Rad Laboratories, s.r.l., Segrate, Italy).

### 2.6 Separation of respiratory complexes and supercomplexes by Blue-Native Page (BN-PAGE) and histochemical staining for in-gel activity

Solubilization of mitochondrial membranes by detergents, BN-PAGE, staining, and densitometric quantification of oxidative phosphorylation complexes was performed essentially as described by Schagger et al. and Silvestri et al. with minor modifications (Schägger and von Jagow, 1991; Silvestri et al., 2018). Briefly, the mitochondria containing sediment was suspended in 50 mM NaCl, 50 mM imidazole, pH 7.0 and solubilized with 10% dodecylmaltoside (for solubilization of individual respiratory chain complexes) or digitonin (4 g/g protein, for solubilization of respiratory chain supercomplexes). After the electrophoretic, enzymatic colorimetric reactions were performed essentially as reported by others (Zerbetto et al., 1997). Complex I activity was determined by incubating the gel slices with 2 mM Tris–HCl, pH 7.4, 0.1 mg/mL NADH, and 2.5 mg/mL nitro blue tetrazolium (NTB) at room temperature. Complex II activity was evaluated after incubating the gel slices at room temperature in a 100 mM Tris/glycine buffer at pH 7.4 containing 1 mg/mL NTB and 1 mM sodium succinate. Complex IV activity was estimated by incubating BN-PAGE gels with 5 mg 3,3′- diaminobenzidine tetrahydrochloride (DAB) dissolved in 9 mL phosphate buffer (0.05 M, pH 7.4), 1 mL catalase (20 μg/mL), 10 mg cytochrome c, and 750 mg sucrose. After gel scanning, the areas of the bands were expressed as absolute values (arbitrary units).

### 2.7 Preparation of mitochondrial lysates from the liver

The mitochondrial pellet was resuspended in RIPA buffer containing 150 mM NaCl, 1.0% Triton X-100, 0.5% sodium deoxycholate, 0.1% SDS, 50 mM Tris, pH 8.0) supplemented with 1 mM Na_3_VO_4_, 1 mM PMSF, and 1 mg/mL Leupeptin. The homogenate was left on ice for 1 h, and shaken every 10 min. The protein concentrations of the homogenates were determined as described in the previous sections.

### 2.8 Preparation of total lysates from the liver, electrophoreses, and immunoblotting

Liver samples were homogenized in Lysis Buffer containing 20 mM Tris-HCl (pH 7.5), 150 mM NaCl, 1 mM EDTA, 1 mM EGTA, 2.5 mM Na_2_H_2_P_2_O_7_, 1 mM b-CH_3_H_7_O_6_PNa_2_, 1 mM Na_3_VO_4_, 1 mM PMSF, 1 mg/mL Leupeptin, and 1% (v/v) Triton X-100 (Sigma- Aldrich, St. Louis, MO, United States) using an UltraTurrax homogenizer, and then centrifuged at 16,000×g in a Beckman Optima TLX Ultracentrifuge (Beck-man Coulter S.p.A., Milan, Italy) for 15 min at 4°C. The supernatants were then ultracentrifuged at 40,000×g in a Beckman Optima TLX ultracentrifuge for 20 min at 4°C. A DC method developed by Bio-Rad was used to determine the protein concentrations in the supernatants of centrifuged lysates (Bio-Rad Laboratories, s.r.l., Segrate, Italy). Electrophoresis on SDS-PAGE gels and Western blot analysis were performed essentially as described by Petito et al. and (Petito et al., 2021; Senese et al., 2019). Briefly, total lysates containing 30 μg proteins were diluted in an equal volume of 5X Laemmli’s reducing sample buffer (62.5 mM Tris pH 6.8, 10% glycerol, 2% SDS, 2.5% pyronin, and 200 mM di-thiothreitol) and incubated at 95°C for 5 min. The samples were loaded into each lane and electrophoresed on SDS-PAGE gels in Tris/glycine/SDS running buffer (pH 8.3). At the end of the run, the proteins were transferred to a nitrocellulose membrane. Then they were blocked with 5% (w/v) nonfat dry milk (Cat. no. 42590.01 Serva) (in TBS-T). Subsequently, they were probed with the following primary antibodies: anti- Total OXPHOS antibody (1:500; ab110413; Abcam), anti-PGC1α (1:1,000; AB3242; Millipore), anti-NRF1 (1:20,000; ab34682; Abcam), anti-TFAM (1:1,000; sc-166965; Santa Cruz Bio-technology), anti- OPA1 (1:1,000; ab157457; Abcam), anti- DRP1 (1:1,000; #5391; Cell Signaling), anti- MFN2 (1:1,000; ab56889; Abcam), anti- MFN1 (1:1,000; ab126575; Abcam), anti- APEX1 (1:1,000; NB100-116; Novus Biologicals), anti- OGG-1 (1:1,000; NB100-106; Novus Biologicals), anti- POL-γ (1:1,000; sc-5931; Santa Cruz Biotechnology), anti- PARKIN (1:1,000; #4211; Cell Signaling), anti- PINK1 (1:1,000; ab186303; Abcam), anti- AMBRA1 (1:1,000; #24907; Cell Signaling); anti- FUNDC1 (1:1,000; A16318; ABclonal); anti-P-ULK (Ser555) (1:1000; #5869; Cell Signaling); anti- ULK1 (1:1,000; #8054; Cell Signaling), anti- LC3B (1:1,000; sc-376404; Santa Cruz Biotechnology), anti- SQSTM1/p62 (1:1,000; #5114; Cell Signaling), anti- CATALASE (1:750; C0979; Sigma Aldrich), anti- SOD2 (1,000; ab68155; Abcam), anti- GPX1 (1:1,000; GTX03346; GeneTex), anti- P-PERK (Thr980) (1:1,000; #3179; Cell Signaling), anti- PERK (1:1,000; #3192; Cell Signaling), anti- P-eiF2α (1:1,000; #3398; Cell Signaling), anti-eiF2α (1:1,000; #2103; Cell Signaling), anti- LONP1 (1:1,000; A4293; ABclonal), anti- CLPP (1:1,000; A3214; ABclonal), anti- TRAP1 (1:1,000; A2748; ABclonal), anti- ATF5 (1:1,000; A3563; ABclonal), anti- ATF4 (1:1,000; A18687¸ABclonal), anti- CHOP (1:1,000; A0221; ABclonal), anti- VDAC (1:1,000; GTX114187; GeneTex) and B-ACTIN (1:1,000; bs-0061R; Bioss Antibodies). The secondary antibodies used were Goat Anti-Rabbit IgG (HRP) (1:4,000; ab97051; Abcam) and Goat Anti- Mouse IgG (HRP) (1:4,000; ab97023; Abcam). The chemiluminescence signal was detected using the Chemi Doc XRS+ (Biorad). For densitometric analysis, the software Image Lab 6.0.1 (Hercules, CA, United States of America) was used.

### 2.9 RNA isolation and quantitative real time PCR

Liver samples (10 mg) were homogenized using a polytron in QIAzol lysis buffer (Cat. No. 79306 QIAGEN). RNA was extracted by the miRNeasy mini kit (Cat. no. 217084 QIAGEN). Total RNA (1 μg) was used to synthesize cDNA strands in a 20-μL reaction volume using the SuperScript IV Reverse Transcriptase for RT-PCR (Cat. no. 18090010 Invitrogen). Quantitative RT-PCR (QRT-PCR) was conducted with 50 nM gene specific primers, IQ SYBR Green supermix (Bio-Rad), and cDNA samples (2 μL) in a total volume of 25 μL. A melting curve analysis was completed following amplification from 55°C to 95°C to ensure product identification and homogeneity. The mRNA expression levels were repeated in triplicate and were normalized to a reference gene (b-actin and Gapdh stable under specific experimental conditions) by using the 2^−ΔΔCT^ method. PCR primers were designed by using the Primer three program (Untergasser et al., 2012), synthesized and verified by sequencing at Eurofins Genomics (Ebersberg, Germany).

The primers used were as follows:

Nrf1 (Forward: 5′-GCC​GTC​GGA​GCA​CTT​ACT-3'; Reverse: 5′-CTG​TTC​CAA​TGT​CAC​CAC​C-3′); Nrf2 (Forward: 5′- CTG​AAC​TCC​TGG​ACG​GGA​CTA-3’; Reverse: 5′- CGG​TGG​GTC​TCC​GTA​AAT​GG); Opa1 (Forward: 5′-GAT​GAC​ACG​CTC​TCC​AGT​GA-3'; Reverse: 5′TCG​GGG​CTA​ACA​GTA​CAA​CC-3′); Mfn1 (Forward: 5′-CCC​TGT​CTC​GAA​AAA​CCA​AA-3’; Reverse: 5′-ACT​CCA​GGC​ATT​TGT​CAT​CC); Mfn2(Forward: 5′-GCC​AGC​TTC​CTT​GAA​GAC​AC-3'; Reverse: 5′-GCA​GAA​CTT​TGT​CCC​AGA​GC-3′); Tfam (Forward: 5′CCA​CAT​GCT​TTC​TTG​GGT​TT-3'; Reverse: 5′-TGC​TGT​GGT​TTC​CCA​GTG​TA-3′); Pgc1α (Forward: 5′-TTC​TGG​GTG​GAT​TGA​AGT​GGT​G-3'; Reverse: 5′-TGT​CAG​TGC​ATC​AAA​TGA​GGG​C-3′); Drp1(Forward: 5′-GGG​CAC​TTA​AAT​TGG​GCT​CC-3'; Reverse: 5′-TGT​ATT​CTG​TTG​GCG​TGG​AAC-3′); Ape1 (Forward: 5′-CTA​AGG​GCT​TTC​GTC​ACA​GC-3'; Reverse: 5′-GAG​ACT​TTT​AGC​GGG​CAC​TG-3′); Ogg1 (Forward: 5′-GAT​TGG​ACA​GTG​CCG​TAA-3'; Reverse: 5′-GGA​AGT​GGG​AGT​CTA​CAG-3′); Pol-γ (Forward: 5′- AGT​TGA​AAG​CCA​TGG​TGC​AG -3'; Reverse: 5′-CAA​GTA​CAG​CTG​CGA​TCC​AC -3′); b-actin (Forward: 5′-CAA​CGG​CTC​CGG​CAT​GTG​C-3'; Reverse: 5′-CTC​TTG​CTC​TGG​GCC​TCG-3′); Gapdh (Forward: 5′-GTC​GTG​GAT​CTA​ACG​TGC​C-3'; Reverse: 5′-GAT​GCC​TGC​TTC​ACC​ACC-3′).

### 2.10 Statistical analysis

Comparisons were performed using GraphPad Prism 8.0.1. The values were compared by Student’s t-test for between-group comparisons. Differences with *p* < 0.05 were considered statistically significant. All data were expressed as the mean ± SEM from at least five independent animals (n = 5).

## 3 Results

### 3.1 The lack of CB1 affects phenotypic parameters and the functional/structural organization of the respiratory chain in mice

**FIGURE 1:**
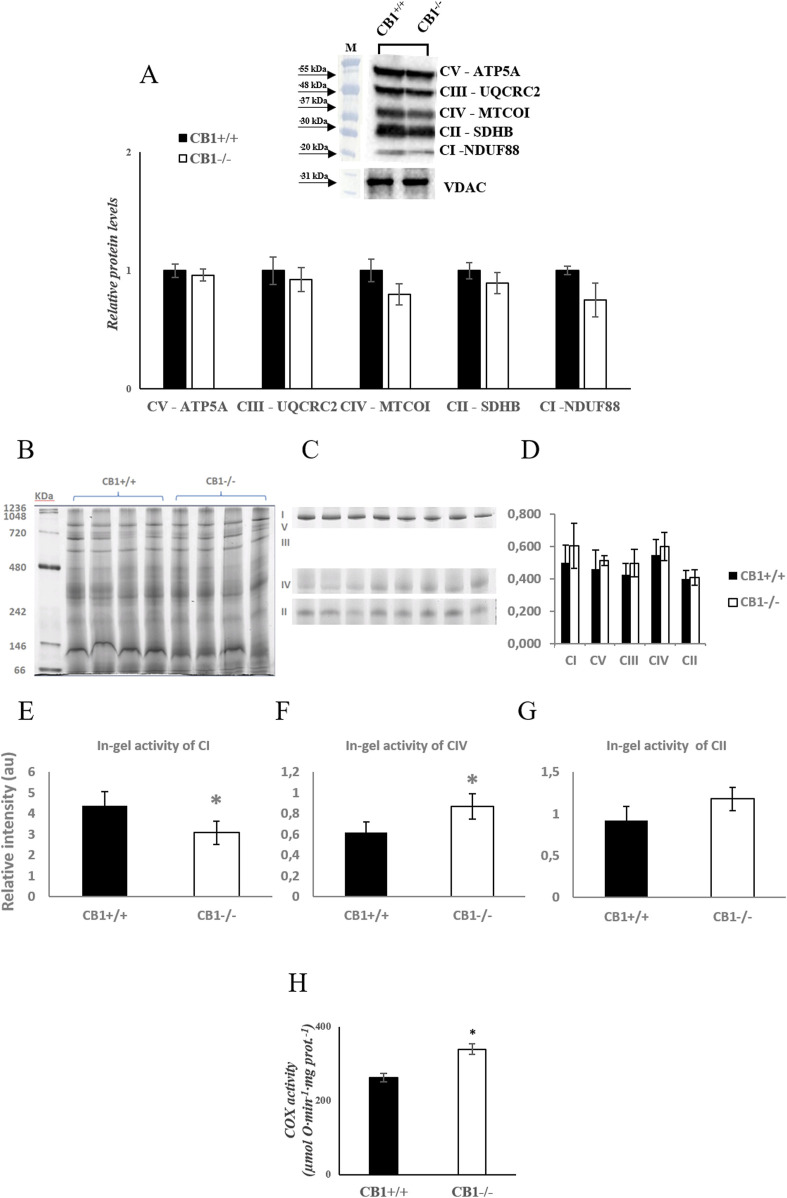
CB1 deletion affects functional/structural organization of the respiratory chain. **(A)** Representative immunoblots of CI–CV respiratory chain complex protein levels in liver mitochondria of CB1^+/+^ and CB1^−/−^ mice. The protein level was normalized to that of VDAC. Histograms show the results of densiometric analysis of immunoblots. BN-PAGE-based analysis of individual respiratory complexes from dodecylmaltoside-solubilized crude mitochondria from the liver of CB1^+/+^ and CB1^−/−^ mice. **(B)** Representative image of a Coomassie blue stained BN-PAGE gel. Bands characteristic of individual OXPHOS complexes are recognizable. Molecular weights of standard proteins and the relative position of the respiratory complexes (I–V) are indicated. **(C)** Representative images of histochemical staining of complex I (I), complex IV (IV), and complex II (II) in-gel activity. **(D)** Densitometric quantification of the blue bands corresponding to individual complexes (arbitrary units, a.u). **(E–G)** Densitometric quantification of bands corresponding to individual in-gel activity of complex I **(E)**, IV **(F)**, and II **(G)** (arbitrary units, a.u). **(H)** Activity of Cytochrome C Oxidase in liver mitochondria of CB1^+/+^ and CB1^−/−^ mice. Protein extracts were prepared for each animal, and each individual was assessed separately. Protein load was 15 μg/lane. All values are represented as mean ± SEM; (n = 5/group). Student’s t-test was used for statistical analysis. *p* < 0.05 was considered significant. *p < 0.05 vs. CB1^+/+^. Abbreviations: COX, Cytochrome C Oxidase.

Body weight, weight of liver, skeletal muscle, and fat pads as phenotypic parameters of CB1^+/+^ and CB1^−/−^ male mice were measured. The results showed that body weight and liver weight were significantly lower in CB1^−/−^ mice compared to CB1^+/+^ mice as was the weight of several fat depots (Table 1). In addition, in the CB1^−/−^ mice, we found a significant increase in brown adipose tissue (BAT) mass (Table 1). Western blot analysis revealed that in hepatic mitochondria of CB1^−/−^ mice the protein levels of OXPHOS complexes were unchanged when compared to mitochondria isolated from CB1^+/+^ (Figure 1A). This result was confirmed by BN-PAGE analysis of OXPHOS complexes obtained from liver mitochondria of CB1^+/+^ and CB1^−/−^ mice. In Figure 1B, the five major Coomassie blue-stained bands represent the individual OXPHOS complexes (I-V). As far as it concerns their amount, densitometric analysis revealed that there were no significant differences between mitochondria from CB1^+/+^ and CB1^−/−^ mice (Figure 1D). In-gel activities of the purified OXPHOS complexes I, II, and IV were estimated by an examination of complex-specific enzymatic colorimetric reactions. The stained enzymatic activities of the assayed complexes were localized specifically to a single band (Figure 1C). In the absence of significant differences in complex II in-gel activity (+28% vs. CB1^+/+^), CB1^−/−^ mice showed reduced in-gel activity of complex I (−30% vs CB1^+/+^) and increased in-gel activity of complex IV (+40% vs. CB1^+/+^) (Figures 1E–G). In addition, we measured specific COX activity in mitochondria isolated from the liver of CB1^+/+^ and CB1^−/−^ mice. COX activity was significantly increased in the liver of CB1^−/−^ mice (Figure 1H).

**TABLE 1:** Effect of CB1 deletion on phenotypic parameters. Body weight (g), Liver weight (g), Skeletal muscle weight (g), vWAT weight (g), sWAT weight (g), eWAT weight (g), and BAT weight (g) of CB1^+/+^ and CB1^−/−^ mice. Values are represented as mean ± SEM; (n = 5/group). *p < 0.05 vs. CB1^+/+^ (Student’s t-test). Abbreviations: vWAT, visceral White Adipose Tissue; sWAT, subcutaneous White Adipose Tissue; eWAT, epididymal White Adipose Tissue; BAT, Brown Adipose Tissue.

Parameters	CB1^+/+^	CB1^−/−^
Body Weight (g)	51.8 ± 0.4	47.5 ± 0.5*
Liver (g)	4.7 ± 0.1	3.9 ± 0.1*
Skeletal Muscle (g)	0.4 ± 0.03	0.3 ± 0.03
Visceral WAT (g)	0.9 ± 0.04	0.6 ± 0.02*
Subcutaneous WAT (g)	0.9 ± 0.05	0.7 ± 0.01*
Epididymal WAT (g)	2.2 ± 0.05	1.8 ± 0.09*
BAT (g)	0.4 ± 0.03	0.7 ± 0.1*

**FIGURE 2:**
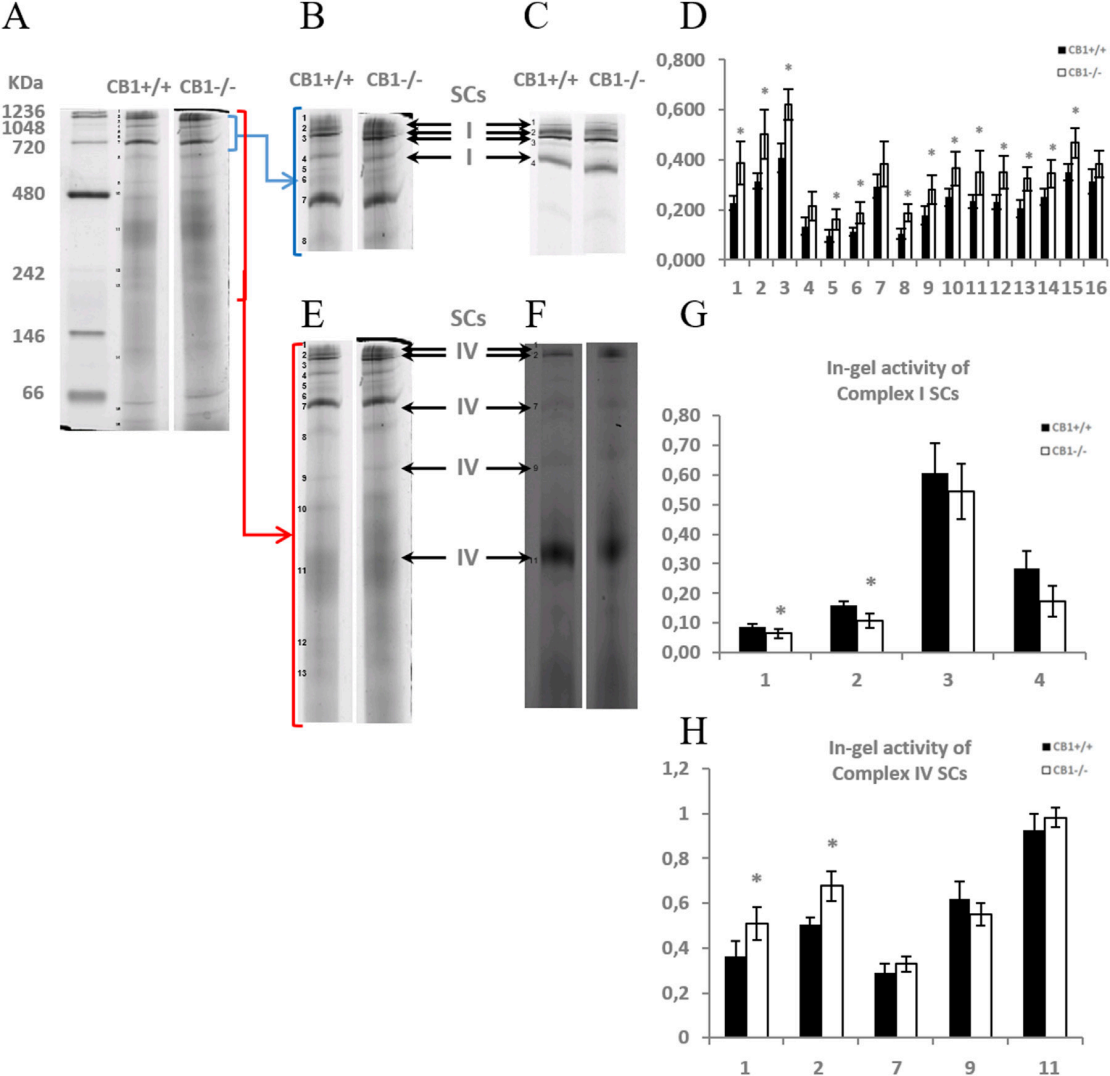
CB1 deletion affects the functional/structural organization of the respiratory chain. Blue native PAGE based analysis of digitonin-solubilized crude mitochondria from the liver of CB1^+/+^ and CB1^−/−^ mice. **(A)** Representative image of a Coomassie blue stained BN-PAGE gel. Bands characteristic of OXPHOS supercomplexes are recognizable in all the experimental groups and highlighted in **B, E** (bands of obtained OXPHOS supercomplexes were numbered from 1 (top of the gel) to 16 (bottom of the gel). **(C, F)** Representative images of histochemical staining of in-gel activity of complex I (I) **(C)** and complex IV (IV) **(F)** supercomplexes (arrows). **(D)** Densitometric quantification of the blue bands corresponding to individual supercomplexes. **(G, H)** Densitometric quantification of bands corresponding to in-gel activity of supercomplex I **(G)** and IV **(H)**. **(D, G, H)** *x*-axis: equal numbers correspond to the same bands in **(A–C, E, F)**. Protein extracts were prepared for each animal, and each individual was assessed separately. Protein load was 15 μg/lane. Values are represented as mean ± SEM; (n = 5/group). Student’s t-test was used for statistical analysis. *p* < 0.05 was considered significant. *p < 0.05 vs. CB1^+/+^.

To elucidate whether the knockout of the CB1 gene also alters the functional/structural organization of the respiratory chain in terms of the assembly and activity of OXPHOS supercomplexes, we extracted liver mitochondria using the mild detergent digitonin (digitonin/protein ratio of 4 g/g), since this extensively retains inner mitochondrial membrane supercomplexes and the resulting proteins were resolved by BN-PAGE and subsequently assayed for complex I- and complex IV- in-gel activities (Figure 2). Complex I activity resulted to be present in four high molecular mass supercomplexes (SCI1-4), within the mass range 720–1236 KDa (Figures 2A–C), the highest activity corresponding to the band SCI3 (Figure 2G). As far as it concerns complex IV, the activity of such enzyme resulted to be present in five SCs (SCIV1, 2, 7, 9,11), within the mass range 242–1236 KDa (Figures 2A–F), the highest activity corresponding to the lowest molecular mass band, namely, SCIV11 (Figure 2H). In some heavier SCs, CI and CIV activity coexisted, specifically in SCI1 (SCIV1) and SCI2 (SCIV2). When comparing the BN-PAGE blue-colored supercomplex profiles between CB1^+/+^ and CB1^−/−^ mice, a general up-representation of respiratory SCs was observed in mitochondria from CB1^−/−^ mice (Figure 2D). The knockout of CB1 was associated with a significant reduction of the in-gel activity of CI detected in the SC bands SCI1 and SCI2 and a significant increase of the in-gel activity of CIV detected in SCIV1 and SCIV2 (Figures 2G, H). What was observed for in-gel activity of CI- and CIV- containing SCs resulted in parallel to what was observed for the individual respiratory complexes.

### 3.2 The lack of CB1 affects antioxidant defense status

**FIGURE 3:**
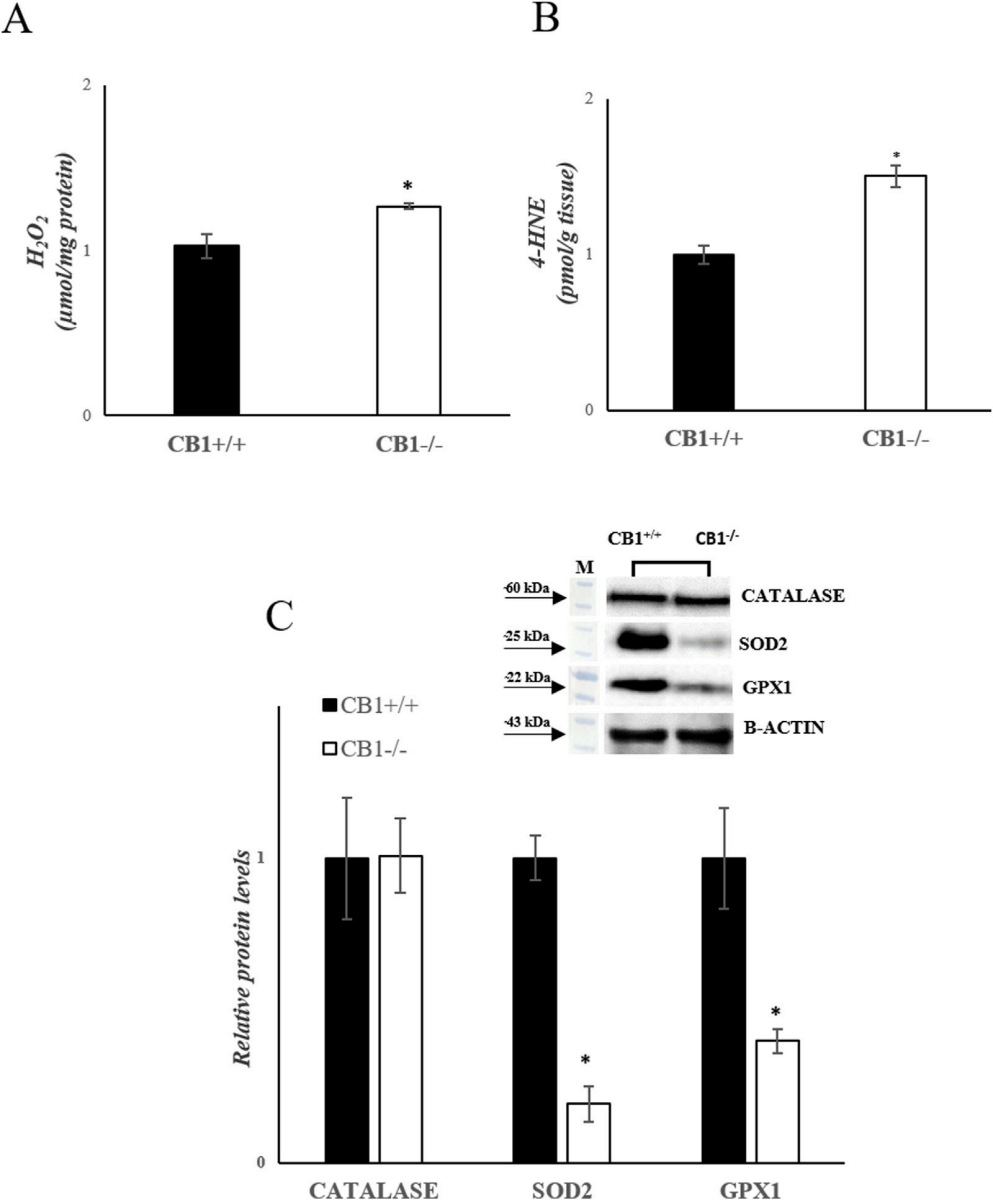
CB1 deletion affects antioxidant defense status. **(A)** H_2_O_2_ levels (μmol/mg protein) in the liver of CB1^+/+^ and CB1^−/−^ mice. **(B)** 4-HNE levels (pmol/g tissue) in the liver of CB1^+/+^ and CB1^−/−^ mice. **(C)** Representative immunoblots of CATALASE, SOD2, and GPX1 in the liver of CB1^+/+^ and CB1^−/−^ mice. The protein level was normalized to that of B-ACTIN. Histograms show the results of densiometric analysis of immunoblots. All values are represented as mean ± SEM; (n = 5/group). Student’s t-test was used for statistical analysis. *p* < 0.05 was considered significant. *p < 0.05 vs. CB1^+/+^. Abbreviations: H_2_O_2_, hydrogen peroxide; 4-HNE, 4-Hydroxynonenal; CATALASE, Catalase; SOD2, Superoxide Dismutase 2; GPX1, Glutathione Peroxidase 1.

The increase in hepatic oxidative capacity affects ROS production. Mitochondrial hydrogen peroxide (H_2_O_2_) release, an indirect index of mitochondrial superoxide production, was significantly increased in the liver of CB1^−/−^ mice when compared with CB1^+/+^ (Figure 3A). We also measured the levels of 4-Hydroxy-2-Nonenal (4-HNE) in liver homogenates. 4-HNE is a highly reactive aldehyde produced as a consequence of lipid peroxidation by ROS and it is considered one of the most important markers of oxidative stress. Our results showed that 4-HNE levels were significantly increased in CB1^−/−^ mice when compared with CB1^+/+^ animals (Figure 3B). Antioxidant enzymes such as Catalase (CATALASE), Superoxide Dismutase 2 (SOD2) and Glutathione Peroxidase 1 (GPX1) play a key role in protecting cells from oxidative damage by catalyzing the dismutation of superoxide anion to hydrogen peroxide. As reported in Figure 3C, the protein levels of SOD2 and GPX1 were significantly decreased in the liver of CB1^−/−^ mice when compared with CB1^+/+^ animals while protein levels of CATALASE were not significantly different among the groups (Figure 3C). The observed differences between CB1^+/+^ and CB1^−/−^ mice suggest oxidative damage in the liver of the CB1^−/−^ animals.

### 3.3 The lack of CB1 affects mitochondrial quality control (MQC)

**FIGURE 4:**
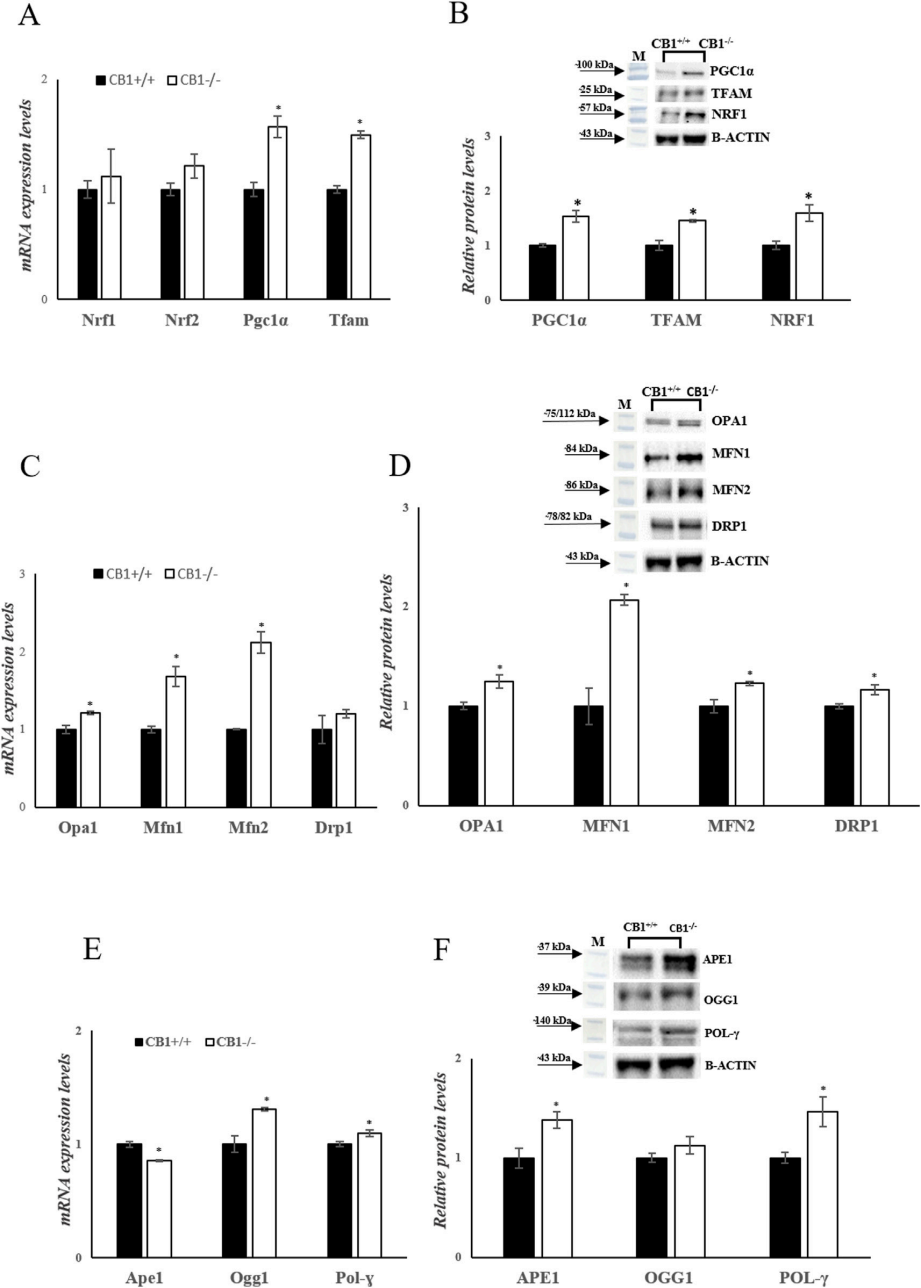
CB1 deletion affects biogenesis, fission, fusion, and mtDNA repair. **(A), (C)** and **(E)** mRNA expression of Nrf1, Nrf2, Pgc1α, Tfam, Opa1, Mfn1, Mfn2, Drp1, Ape1, Ogg1, Pol-γ in the liver of CB1^+/+^ and CB1^−/−^ mice. The mRNA level was normalized to that of B-actin and Gapdh. **(B), (D)** and **(F)** Representative immunoblots of PGC1α, TFAM, NRF1, OPA1, MFN1, MFN2, DRP1, APE1, OGG1, POL-γ in the liver of CB1^+/+^ and CB1^−/−^ mice. The protein level was normalized to that of B-ACTIN. Histograms show the results of densiometric analysis of immunoblots. All values are represented as mean ± SEM; (n = 5/group). Student’s t-test was used for statistical analysis. *p* < 0.05 was considered significant. *p < 0.05 vs. CB1^+/+^. Abbreviations: PGC1α, Peroxisome Proliferative-Activated Receptor Gamma Coactivator 1α; NRF1, Nuclear Respiratory Factor 1; NRF2, Nuclear Respiratory Factor 2; TFAM, Mitochondrial Transcription Factor A; MFN1, Mitofusin 1; MFN2, Mitofusin 2; OPA1, Optic Atrophy 1; DRP1, Dynamin-Related Protein 1; POL-γ, DNA Polymerase γ; OGG1, 8-Oxoguanine Glycosylase 1; APE1, Apurinic/Apyrimidinic Endonuclease 1.

To investigate mitochondrial features in the mouse model of CB1 absence, key players of mitochondrial quality control mechanisms were evaluated. Changes in the expression levels of Peroxisome proliferative-activated receptor gamma coactivator 1α (Pgc1α), Nuclear respiratory factor 1 and 2 (Nrf1, Nrf2), and Mitochondrial Transcription Factor A (Tfam), all considered master regulators of mitochondrial biogenesis were evaluated. In CB1^−/−^ mice, Pgc1α and Tfam mRNA expressions were significantly increased compared to CB1^+/+^ mice (Figure 4A). Nrf1 transcription was increased by 1.12-fold (vs CB1^+/+^) but did not reach statistical significance (Figure 4A). Western blot analysis revealed that protein levels of PGC1α, NRF1, and TFAM were significantly increased in CB1^−/−^ mice vs CB1^+/+^ (Figure 4B). Mitofusin 1 (Mfn1), Mitofusin 2 (Mfn2) and Optic atrophy 1 (Opa1), which are located in the outer and the inner mitochondrial membranes respectively, are GTPase proteins that regulate mitochondrial fusion. On the other hand, Dynamin-related protein 1 (Drp1) controls mitochondrial fission. To further understand how mitochondrial dynamics are affected by CB1 deficiency, we next examined the expression of these above-mentioned markers intrinsically associated with mitochondrial dynamics. As reported in Figures 4C, D, the expression levels of Opa1, Mfn1and Mfn2 and the protein levels of OPA1, DRP1, MFN1 and MFN2 were significantly increased in the liver of CB1^−/−^ mice compared to the wild-type animals highlighting an increase in mitochondrial dynamic (Figures 4C, D). Mitochondrial homeostasis is also affected by the Base Excision Repair pathway (BER). As BER is critical for maintaining mtDNA integrity, we analyzed the expression levels of its components. Our results showed that the expression levels of DNA Polymerase γ (Pol-γ), 8-Oxoguanine glycosylase 1 (Ogg1), Apurinic/apyrimidinic endonuclease 1 (Ape1), and the protein levels of POL-γ and APE1 were significantly increased in CB1^−/−^ mice compared to CB1^+/+^ animals (Figures 4E, F). The protein levels of OGG1 were increased by about 1.12% in CB1^−/−^ mice but did not reach statistical significance (Figure 4F). These data suggest that the lack of CB1 increases mtDNA repair by increasing the BER pathway.

**FIGURE 5:**
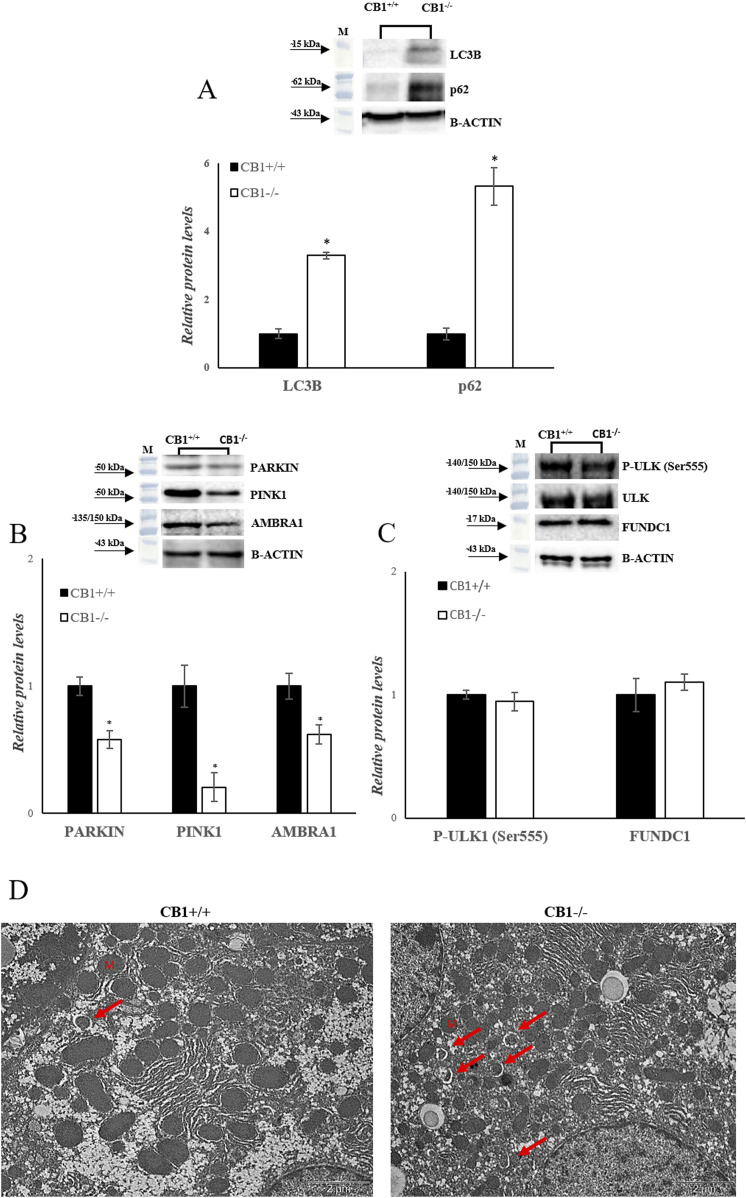
CB1 deletion affects autophagy/mitophagy processes. **(A)**, **(B)** and **(C)** Representative immunoblots of LC3B, p62, PARKIN, PINK1, AMBRA1, P-ULK (Ser555), FUNDC1 in the liver of CB1^+/+^ and CB1^−/−^ mice. Histograms show the results of densiometric analysis of immunoblots. The protein level was normalized to that of B-ACTIN and/or to the total forms for phosphorylated proteins. **(D)** Representative images of liver ultrastructure under TEM in CB1^+/+^ and CB1^−/−^ mice. Scale bars: 2 μm. Red arrows pointed towards autophagosomes. All values are represented as mean ± SEM; (n = 5/group). Student’s t-test was used for statistical analysis. *p* < 0.05 was considered significant. *p < 0.05 vs. CB1^+/+^. Abbreviations: LC3B, Microtubule-Associated Protein 1 Light Chain 3 Isoform B; SQSTM1/p62, Sequestosome 1; PARKIN, Parkin; PINK1, PTEN-Induced Kinase 1; AMBRA1, Activating Molecule in Beclin1-Regulated Autophagy; ULK, Unc-51 Like Autophagy Activating Kinase 1; FUNDC1, FUN14 Domain Containing 1.

Besides mitochondrial biogenesis, dynamics, and mtDNA repair, mitophagy is another important component for mitochondrial quality maintenance. In mammalian cells, mitophagy is mediated by key proteins: the Outer mitochondrial membrane kinase Pink1 (PTEN-induced kinase 1), cytosolic Parkin (E3 ubiquitin ligase), and Activating Molecule in Beclin1-Regulated Autophagy (AMBRA1). CB1^−/−^ mice showed a significant reduction in PARKIN, PINK1 and AMBRA1 protein levels, as shown in Figure 5B (Figure 5B). In addition to Pink1/PARKIN-dependent pathway exits a Pink1/PARKIN-independent pathway. In this pathway LC3 directly binds with the outer mitochondrial membrane proteins as FUNDC1 and ULK1 via the LC3B interacting region (Terešak et al., 2022; Wu et al., 2014). CB1^−/−^ mice showed unchanged levels of P-ULK1 and FUNDC1 as shown in Figure 5C (Figure 5C). Furthermore, sequestosome 1 (SQSTM1/p62) and microtubule-associated protein one light chain three isoform B (LC3B), considered autophagy markers, were measured. SQSTM1/p62 elevated levels inhibit autophagy, whereas their decreased levels activate it (Dupont et al., 2014). Significantly increased protein levels of LC3B and SQSTM1/p62 were observed in the liver homogenates of CB1^−/−^ mice compared to the wild-type animals (Figure 5A). The TEM analysis revealed that CB1^−/−^ mouse hepatocytes did not present significantly altered features with respect to CB1^+/+^ ones in terms of, glycogen and lipid depots, outer and inner cell membranes damage, and mitochondria morphology alteration, indicating the possible trigger of compensative mechanisms to maintain CB1^−/−^ hepatocyte ultrastructure. However, it should be mentioned that in CB1^−/−^ hepatocytes some early mitophagic vesicles were observable that we did not find in CB1^+/+^ hepatocytes (Figure 5D). All together, these results could indicate that in the absence of CB1, there is an initiation of the mitophagy process, which consists in the formation of a phagophore (Figure 5D), but its further growth and transformation into an autophagosome is inhibited, suggesting an impaired autophagic flux in the liver of CB1^−/−^ mice.

**FIGURE 6:**
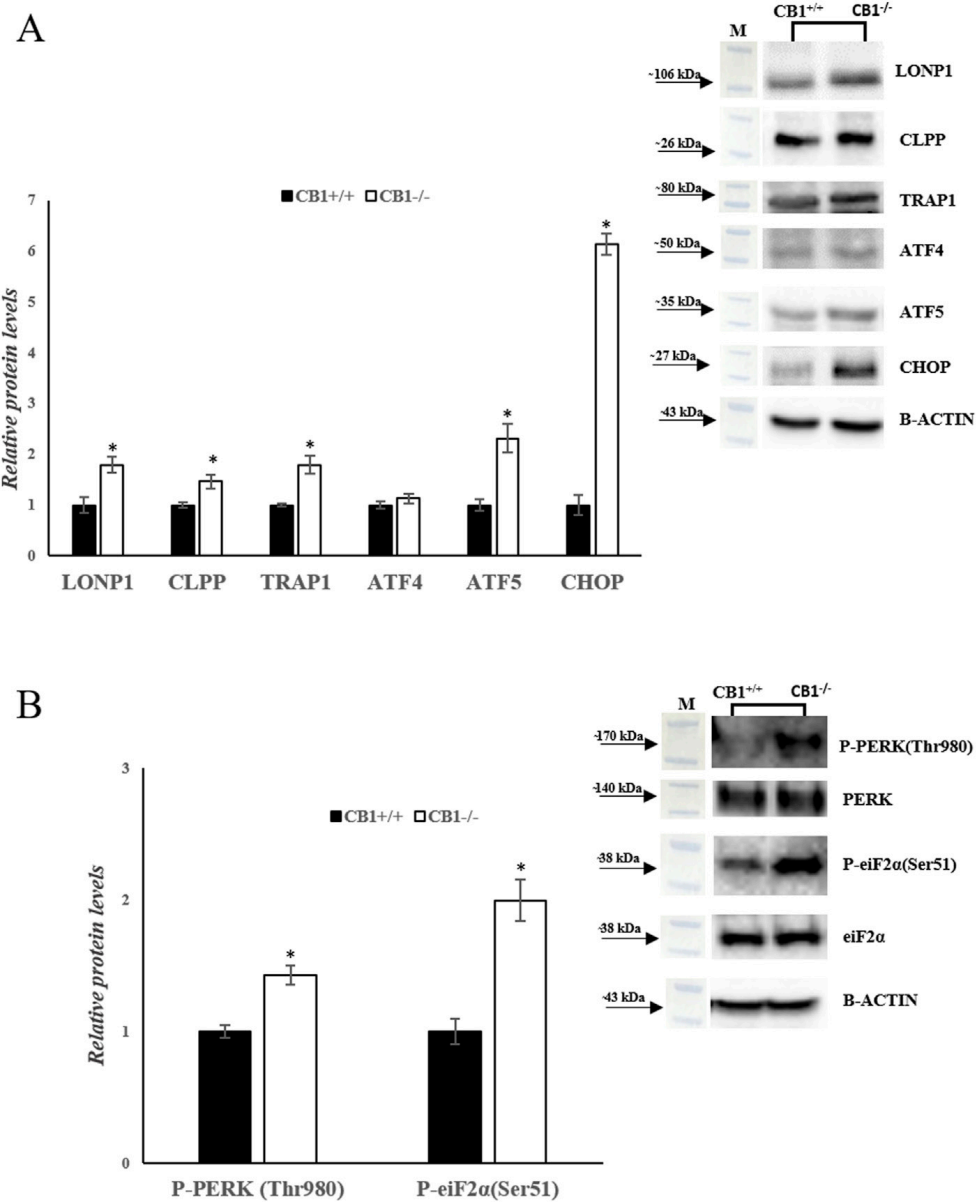
CB1 deletion affects UPR^mt^ and UPR^ER^ processes. **(A)** and **(B)** Representative immunoblots of LONP1, CLPP, TRAP1, ATF4, ATF5, CHOP, P-PERK(Thr980), P-eiF2α(Ser51) in the liver of CB1^+/+^ and CB1^−/−^ mice. The protein level was normalized to that of B-ACTIN and/or to the total forms for phosphorylated proteins. Histograms show the results of densiometric analysis of immunoblots. All values are represented as mean ± SEM; (n = 5/group). Student’s t-test was used for statistical analysis. *p* < 0.05 was considered significant. *p < 0.05 vs. CB1^+/+^. Abbreviations: LONP1, Lon Peptidase 1; CLPP, Caseinolytic Mitochondrial Matrix Peptidase Proteolytic Subunit; TRAP1, TNF Receptor Associated Protein 1; ATF4, Activating Transcription Factor 4; ATF5, Activating Transcription Factor 5; CHOP, C/EBP Homologous Protein; PERK, Protein Kinase R (PKR)-Like Endoplasmic Reticulum Kinase; eiF2α, Eukaryotic Translation Initiation Factor 2A.

Besides mitochondrial biogenesis, dynamics, mitophagy, and mtDNA repair, UPR^mt^ also contributes to mitochondrial homeostasis. We analyzed a canonical UPR^mt^ axis. The protein levels of LONP1 and CLPP were increased significantly in CB1^−/−^ mice. In addition, the protein levels of TRAP1, ATF4, ATF5, and CHOP were significantly increased in CB1^−/−^ mice when compared to the control group (Figure 6A). Finally, since UPR^mt^ and UPR^ER^ signaling pathways are interconnected to maintain cellular homeostasis under stress conditions, we measured the phosphorylation levels of PERK and Eukaryotic translation initiation factor 2A (eiF2α). The phosphorylation of both these proteins was significantly increased in the liver of CB1^−/−^ mice when compared to the control group (Figure 6B). This condition suggests that UPR^ER^ is activated in CB1^−/−^ mice.

## 4 Discussion

Mitochondria exhibit high adaptability and dynamism, playing crucial roles in cellular metabolism and stress responses. Serving as a center for biochemical processes such as Adenosine Triphosphate (ATP) production, synthesis of fatty acids, generation of intracellular ROS, thermogenesis, and calcium homeostasis, mitochondria are recognized as the central regulators of energy metabolism. Therefore, ensuring the balance and proper functioning of mitochondria, known as mitochondrial homeostasis and proteostasis, is vital for overall cellular health. The CB1 are implicated in regulating tissue metabolism not only by influencing mitochondrial activity but also by maintaining mitochondrial integrity.

Studies indicate that CB1 agonists activate CB1 to induce intra-mitochondrial processes important for synaptic transmission (Djeungoue-Petga and Hebert-Chatelain, 2017). Additionally, research suggests that CB1 regulate mitophagy in hippocampal neurons, with CB1^−/−^ mice displaying altered mitochondrial dynamics and reduced mitophagy activity compared to CB1^+/+^ mice (Beji et al., 2020). Furthermore, the blockade of CB1 has been linked to promoting mitochondrial biogenesis through the expression of endothelial Nitric Oxide Synthase (NOS) in white adipocytes, potentially involving 5′AMP-activated protein kinase (AMPK) in this process (Tedesco et al., 2008). On the contrary, stimulation of CB1 has been associated with impairing mitochondrial biogenesis in various tissues such as White Adipose Tissue (WAT), muscle, and liver (Tedesco et al., 2010). This suggests a dual role for CB1 in mitochondrial function, where activation and blockade can have contrasting effects on mitochondrial processes. Moreover, the presence of CB1 on mitochondrial membranes of different cell types underscores their involvement in regulating mitochondrial functions under pato-physiological conditions (Aquila et al., 2010; Bénard et al., 2012; Kamnate et al., 2022; Mendizabal-Zubiaga et al., 2016; Pagano Zottola et al., 2022).

The present research reveals that when CB1 are lacking, various adaptive response mechanisms come into play to preserve mitochondrial function, thereby seeking to ensure liver function.

Our study initially confirms that the absence of CB1 in CB1^−/−^ mice significantly affects body weight regulation, with these mice displaying lower body weight than their wild-type counterparts (Table 1). This result aligns with previous research illustrating the crucial role of CB1 in modulating appetite, food intake, and energy expenditure (Cota et al., 2003). Furthermore, the decrease in body weight observed in CB1^−/−^ mice is accompanied by a substantial reduction of the weight of sWAT, vWAT, and eWAT depots, highlighting the central role of CB1 in the modulation of body adiposity (Rossi et al., 2018). Additionally, CB1^−/−^ mice show a significant increase in BAT weight, suggesting activation of thermogenic mechanisms that might lead to WAT loss in such animals (Table 1). All these changes in fat distribution could be reflected in improved insulin signaling.

Our observations show that the liver, a central organ for metabolic regulation, exhibits adaptative mechanisms in response to CB1 deficiency. CB1^−/−^ mice display a significant increase in liver oxidative capacity, as indicated by increased COX activity (Figure 1). COX is a crucial respiratory complex involved in the mitochondrial electron transport chain, and its increased activity could imply heightened mitochondrial oxygen consumption in CB1^−/−^ mice. The study by Tedesco et al. linking CB1 stimulation to decreased mitochondrial oxygen consumption is in line with this result (Tedesco et al., 2010). Moreover, increased mitochondrial respiration was observed in rats treated with Rimonabant (Flamment et al., 2009), a selective CB1 antagonist with an inverse agonist profile. It was shown that the treatment with Rimonabant led to an increase in total energy expenditure in lean rats, in ob/ob mice, and in rats fed a high-fat and high-carbohydrate diet (Herling et al., 2008; Kunz et al., 2008; Liu et al., 2005). Rimonabant was able to reduce appetite thereby promoting weight loss, improved lipid profiles, and enhanced insulin sensitivity, potentially lowering the risk of type 2 diabetes (Bronander and Bloch, 2007; Thornton-Jones et al., 2006). Additionally, it increases Hypotalamus-Pituitary-Adrenal (HPA) activity and corticosteroid production in food-deprived Zucker rats, suggesting that it activates a stress response (Doyon et al., 2006; Nunn et al., 2009; Steiner et al., 2008). These results initially seemed like a promising approach for managing obesity and metabolic disease in people with poor lifestyle-induced phenotypes. Nevertheless, its severe psychiatric side effects and the broader impact on the ECS led to its withdrawal from the market. Additionally, recent research describes how the activation of mitochondrial CB1 reduces OXPHOS and hampers the metabolism of glucose in mouse astroglia (Jimenez-Blasco et al., 2020).

Increased mitochondrial respiration in CB1^−/−^ mice may result from the influence of CB1 deficiency on the activity and/or expression of other mitochondrial electron transport chain complexes. Our study shows, in the liver of CB1^−/−^ mice, in the absence of significant differences in the protein levels of individual OXPHOS complexes, reduced in-gel activity of complex I and increased in-gel activity of complex IV, paralleled by an even not significant increase of complex II activity (Figure 1). These variations suggest significant changes in the function of the mitochondrial electron transport chain. Complex I is the largest respiratory chain complex, the most sensitive to oxidative damage and one of the sites of ROS production during normal respiration (Koopman et al., 2010; Lenaz et al., 2016; Vinogradov and Grivennikova, 2016). Its inhibition has been reported to elevate oxidative stress, even if differential effects have also been described (Fato et al., 2009; Yin et al., 2021). Likely, reduced activity of complex I observed in CB1^−/−^ mice could be strictly associated with the increased ROS production concomitantly reported in such animals. On the other hand, the reduction of Complex I activity could be a mechanism to prevent oxidative stress induced by ROS release along, ETC. Furthermore, one might speculate that liver cellular metabolism changed in CB1^−/−^ mice, with a reduced use of NADH-substrates and a preference for FADH2-one oxidation. It is possible that this preference for succinate as a substrate will increase the electron transport chain’s rate to support the cell’s energy requirements. However, these speculations suggest further investigation.

Concomitant with the increased oxidative capacity, CB1^−/−^ mice exhibit elevated liver ROS production, mitochondrial H_2_O_2_ release and 4-HNE levels (Figure 3), suggesting a potential pro-oxidative effect resulting from CB1 deficiency within the liver. Furthermore, the protein levels of SOD2 and glutathione GPX1 are significantly decreased in CB1^−/−^ mice (Figure 3). These results are similar to those obtained by Leal et al. who demonstrated that CB1 deficiency in the skin led to an increased production of ROS and a reduction in antioxidant defenses. They postulated that a substantial decline in CB1 expression might contribute to the early aging-like changes observed in diabetes (C Leal et al., 2021). Together, these observations indicate a complex interplay between CB1 signaling and oxidative stress regulation, with potential tissue-specific variations in the response to CB1 deficiency.

Overall, our results suggest that the disruption of CB1 signaling may lead to several changes in the liver, including increased oxidative capacity, reduced activity of complex I, increased activity of complex IV, general upregulation of respiratory supercomplexes, and increased oxidative stress. These changes could have several implications for mitochondrial function and cellular metabolism, including an increased risk of ROS-related damage.

The MQC is a fundamental aspect of cellular homeostasis, ensuring proper mitochondrial function. This intricate network encompasses mechanisms that safeguard mitochondrial health, including mtDNA maintenance, biogenesis, fission, fusion, and mitophagy. Our study provides insights into the impact of CB1 deficiency on MQC within the liver.

The increase in tissue protein levels of POL-γ, OGG1, and APE1 in the livers of CB1^−/−^ mice indicates an enhanced capacity for mitochondrial DNA repair through the BER pathway, suggesting a role for CB1 in maintaining mtDNA integrity and stability. Additionally, the upregulation of mitochondrial biogenesis, fission, and fusion processes in CB1^−/−^ mice, as evidenced by increased protein levels of PGC1α, TFAM, NRF1, NRF2, OPA1, DRP1, MFN1 and MFN2, suggests a dynamic response to the absence of CB1 (Figure 4).

This adaptive mitochondrial plasticity may contribute to enhanced mitochondrial function and metabolic efficiency, potentially compensating for the loss of CB1 signaling. The trigger of compensative mechanisms in CB1^−/−^ hepatocytes can explain also the mainly normal appearance of hepatocyte mitochondrial ultrastructure. In CB1^−/−^ mice, we observe the initiation of mitophagy pathways via LC3B as an adaptive response to counteract ROS-induced damage within the liver. However, a noteworthy finding in our study is the observation of reduced PINK1 and PARKIN levels in CB1^−/−^ mice, key regulators of mitophagy. This intriguing observation suggests that CB1 deficiency triggers the initiation of mitophagy but may concurrently hinder its progression, potentially affecting MQC. Ultrastructural examination of liver tissue corroborated these findings by revealing the presence of phagophores around mitochondria, confirming the initiation of the mitophagic process (Figure 5). In addition, CB1 deficiency does not affect Pink1/PARKIN-independent pathway (Figure 5). Nevertheless, the inhibition of mitophagy does not lead to changes in mitochondrial morphology (Figure 5D), unlike what was noted by Kataoka et al. in hippocampal neurons of adult CB1^−/−^ mice. Their study reveals that diminished mitophagy correlates with the presence of elongated and thinner mitochondria (Kataoka et al., 2020). Recent evidence suggests that oxidative stress, characterized by an imbalance between the production of ROS and the antioxidant defense system, can lead to the accumulation of misfolded proteins in the mitochondria (Muñoz-Carvajal and Sanhueza, 2020). This accumulation triggers the UPR^mt^, the adaptive signaling pathway that aims to restore mitochondrial proteostasis by enhancing protein folding and degrading misfolded proteins (Fiorese and Haynes, 2017). We found that in the liver of CB1^−/−^ mice there is an increase of UPR^mt^ markers.

These findings underscore the vital role of UPR^mt^ activation in preserving mitochondrial homeostasis, complementing its established functions in mitochondrial biogenesis, dynamics, mitophagy, and mtDNA repair. Notably, CB1^−/−^ mice exhibit significantly elevated levels of key proteases such as LONP1 and CLPP involved in the eradication of irreversibly ineffective, damaged and misfolded proteins, suggesting enhanced protein degradation activities essential for maintaining mitochondrial integrity and function (Figure 6). Moreover, CB1^−/−^ mice, compared to the control group, demonstrate increased levels of other transcripts associated with UPR^mt^ including the chaperone TRAP1, and the transcriptional factors ATF4, ATF5 and CHOP which upregulate the gene expression of protease and chaperones (Figure 6).

Furthermore, the interconnection between UPR^mt^ and UPR^ER^, the two signaling pathways involved in maintaining cellular homeostasis under stress, was examined. The phosphorylation levels of P-PERK and P-eIF2α, key components of the UPR^ER^ signaling pathway, were significantly increased in CB1^−/−^ mice compared to the control group (Figure 6). This suggests that UPR^ER^ is activated in CB1^−/−^ mice, indicating a coordinated response to cellular stress involving both UPR^mt^ and UPR^ER^ signaling pathways.

In addition, PINK1/PARKIN mitophagy has been observed to be suppressed in response to oxidative stresses, whereas the UPR^mt^ is activated, suggesting the interplay between these pathways. Indeed, in recent years, multiple signaling pathways have been discovered to regulate mitophagy-mediated mitochondrial degradation. Interestingly, these pathways also activate the UPR^mt^, indicating a potential coordinated control of these two systems responsible for maintaining mitochondrial quality. Evidence shows that under stress conditions, the UPR^mt^ and mitophagy are activated simultaneously and can compensate for each other when one system is insufficient or fails (Wang et al., 2023).

Our findings support a model wherein PINK1/PARKIN mitophagy serves as a final resort quality control mechanism, eliminating mitochondria irreparably damaged beyond the capacity of UPR^mt^. The activation of PINK1/PARKIN mitophagy in situations where UPR^mt^ fails to restore proteostasis is crucial for the preservation of OXPHOS metabolism and likely plays a role in avoiding the protrusion and release of inflammatory mtDNA from severely damaged mitochondria (Uoselis et al., 2023).

In addition, it is important to mention that recent studies have shown that mildly damaged mitochondrial components are processed and disposed in Extracellular Vesicles (EVs) of mitochondrial origin (MDVs) using this alternative degradative route (Sugiura et al., 2014; Terešak et al., 2022). Ramirez et al., in 2022, showed that CBD treatment induced elevated production of MDV suggesting a PINK1-Parkin pathway dependent (Ramirez et al., 2022).

In conclusion, our study highlights the significant impact of CB1 signaling on mitochondrial function particularly in the liver and suggests that a complex adaptive response is triggered by CB1 deficiency to optimize liver function. The absence of CB1 leads to oxidative stress conditions and disrupts mitochondrial proteostasis, triggering the UPR^mt^. The activation of UPR^mt^ in CB1^−/−^ mice underscores its essential role in maintaining mitochondrial homeostasis. We observed elevated levels of key proteases involved in MQC and increased expression of molecular chaperones and transcription factors related to cellular stress responses. Furthermore, our findings reveal a coordinated response to cellular stress, involving both UPR^mt^ and UPR^ER^. In addition, the loss of CB1-mediated signaling could be mitigated by compensatory mechanisms, which allow animals without CB1 to survive. Neurotransmission and various metabolic processes primarily regulated by the CB1, in its absence can be mitigated by other mechanisms, for example, the involvement of CB2 (Bie et al., 2018; Louvet et al., 2011; Munro et al., 1993), and the activation of the G-protein coupled receptor 55 (GPR55) and Vanilloid type 1 receptor (TRPV1) (Aizpurua-Olaizola et al., 2017; Balenga et al., 2011; Chiurchiù et al., 2015; Ryberg et al., 2007; Staton et al., 2008; Yang et al., 2016).

Overall, our study emphasizes the critical importance of CB1 in preserving mitochondrial function and cellular health, shedding light on potential therapeutic targets for conditions involving mitochondrial dysfunction and oxidative stress. Further research is warranted to fully elucidate the intricate mechanisms underlying CB1-mediated mitochondrial regulation and its implications for overall health and disease. However, it is essential to acknowledge that some disparities between our study and the existing literature may arise from distinct experimental conditions. While our investigation focuses on the absence of CB1 in mice without pre-existing liver diseases, other studies assess the effects of CB1 antagonists in animal models with established liver pathologies (Liu et al., 2019; Mboumba Bouassa et al., 2022; Tam et al., 2011). In addition, discrepancies may reflect differences between the effects of short-term modulation of CB1 with the use of pharmacologic agents and the effect of a congenital deficiency of CB1 signaling with activation of compensatory mechanisms in CB1^−/−^ mice.

## Data Availability

The original contributions presented in the study are included in the article/Supplementary Material, further inquiries can be directed to the corresponding author.
